# Critical role of the C5a-activated neutrophils in high-fat diet-induced vascular inflammation

**DOI:** 10.1038/srep21391

**Published:** 2016-02-19

**Authors:** Mizuko Osaka, Shunsuke Ito, Masaki Honda, Yukihiro Inomata, Kensuke Egashira, Masayuki Yoshida

**Affiliations:** 1 Department of Life Science and Bioethics, Graduate School of Medicine, Tokyo Medical and Dental University, Tokyo, Japan; 2 Department of Nutrition and Metabolism in Cardiovascular Disease, Graduate School of Medicine, Tokyo Medical and Dental University, Tokyo, Japan; 3 Department of Transplantation and Pediatric Surgery, Postgraduate School of Medical Sciences, Kumamoto University, Kumamoto, Japan; 4 Department of Cardiovascular Medicine, Graduate School of Medical Sciences, Kyushu University, Fukuoka, Japan

## Abstract

Exceed and chronic high-fat diet (HFD) contributes to the diagnosis and development of atherosclerosis, obesity, and metabolic syndrome. However, the key molecular component(s) triggered by HFD responsible for initiating vascular inflammation remain unknown. We observed that feeding HFD for 4 weeks is sufficient to induce leukocyte recruitment in the femoral artery of wild-type mice. Neutrophil- and monocyte-depletion analyses confirmed the preferential recruitment of neutrophils in these mice. Protein analysis of sera from HFD-fed mice revealed a marked elevation of complement component C5a levels. Exogenous C5a alone induced leukocyte recruitment, which was abolished by a C5a-receptor antagonist. We also examined the role of neutrophil-derived MCP-1 in accumulation of leukocytes in the artery. These results demonstrated a previously unrecognized role for C5a and neutrophils in the early onset of HFD-induced vascular inflammation. Further study may help in elucidating a novel regulatory pathway to control diet-induced inflammation such as that in case of atherosclerosis.

Although excessive intake of fat and cholesterol has been linked to obesity and insulin resistance, the key molecular component(s) triggered by dietary fat responsible for atherosclerosis are remain unknown^1^,^2^,^3^. Recent data suggests that inflammation is involved in this process^4^. A high-fat diet (HFD) increases expression levels of cytokines such as MCP-1, IL-8, MIP-1α, and IL-6^5^,^6^,^7^. However, temporal and spatial details of HFD increases these cytokines are not known. Considering the emergent influence of diet in the development or prevention of atherosclerosis, understanding the initial inflammatory consequences induced by dietary fat intake is of great interest. We therefore examined whether HFD alone induces vascular inflammation manifested as leukocyte recruitment *in vivo*. Although previous studies have demonstrated monocyte adhesion in excised vascular fragments from apolipoprotein E-knockout (ApoE^−/−^) mice, little is known regarding leukocyte recruitment in large arteries such as the femoral artery^8^,^9^. Moreover, genetically engineered mice models of atherosclerosis, such as ApoE^−/−^ or LDL-receptor-knockout mice, exhibited several intrinsic disorders due to the lack of the target gene that may modify the outcomes of HFD. To this end, we conducted a series of studies in which HFD-fed wild-type (wt) mice were used to carefully observe the initial event of vascular inflammation, i.e., the recruitment of leukocyte s in the luminal surface of the artery.

Complement component C5a, which is a pro-inflammatory factor, plays an important role in acute and chronic inflammation, such as in infection, rheumatoid arthritis and cancer^10^,^11^,^12^. However, the involvement of C5a in the up-regulation of C5a and the induction of leukocyte recruitment by C5a in metabolic disorders are not well understood. In this study, we demonstrate a critical role of neutrophils, which are a cellular source of MCP-1 and regulated by upregulation of the complement component C5a in the initial phase of HFD-induced vascular inflammation and subsequent chronic inflammation *in vivo*.

## Results

### HFD increased recruitment of leukocytes to the femoral artery in wt mice

**Figure 1:**
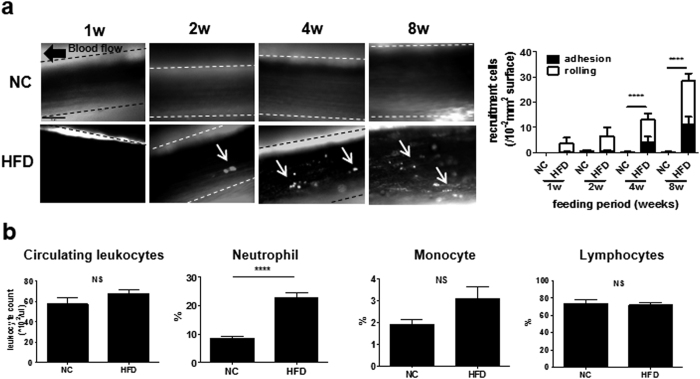
HFD-induced leukocyte recruitment in the femoral artery and neutrophilia of wt mice. (**a**) Leukocyte recruitment in femoral arteries of wt mice fed HFD or NC for 1 (n = 8 and 6, respectively), 2 (n = 8 and 6, respectively), 4 (n = 7 and 8, respectively), or 8 weeks (n = 9 and 8, respectively). Arrowheads show adhesion to the endothelium or rolling leukocytes (left). Bar, 50 μm. Leukocyte recruitment, including leukocyte adhesion and rolling, was significantly increased by HFD for 4 and 8 weeks compared with NC (right). Data are presented as the mean ± SEM. ****P < 0.0001 by two-way ANOVA with Bonferroni’s post test. **(b)** Leukocyte count (n = 7 and 7, respectively) and proportion of neutrophils (n = 11 and 10, respectively), monocytes (n = 8 and 7, respectively), and lymphocytes (n = 4 and 4, respectively) in peripheral blood of wt mice fed NC or HFD for 4 weeks. Neutrophils of mice fed HFD significantly increased compared with those fed NC. Data are presented as the mean ± SEM. ****P < 0.0001 by unpaired; 2-tailed Student’s t test.

First, using intravital microscopy (IVM), we observed leukocyte recruitment in the femoral artery of HFD- or NC-fed wt mice. As shown in Fig. 1a, time-dependent leukocyte recruitment was observed in HFD-fed mice from 1 to 8 weeks (see Supplementary Movie 1,2,4,5,6,7–8). The image analysis revealed that the number of vessel-associated leukocytes was significantly increased at 4 weeks (13.0 ± 3.3/10^4^-μm^2^ vessel surface vs. NC, *P* < 0.0001, n = 7) and 8 weeks (28.4 ± 3.1/10^4^-μm^2^ vessel surface vs. NC, *P* < 0.0001, n = 8) compared with NC (Fig. 1a). Notably, peripheral neutrophil (CD11b^+^Ly-6G^+^) counts were increased when mice were fed HFD (HFD 22.7 ± 1.0%, n = 11; NC 7.7 ± 0.70%, n = 10; *P* < 0.001), although the total leukocyte count did not change (Fig. 1b). The numbers of other cell types, including monocytes and lymphocytes, did not show a marked change.

### Role of neutrophils in HFD-induced leukocyte recruitment in the femoral artery

**Figure 2:**
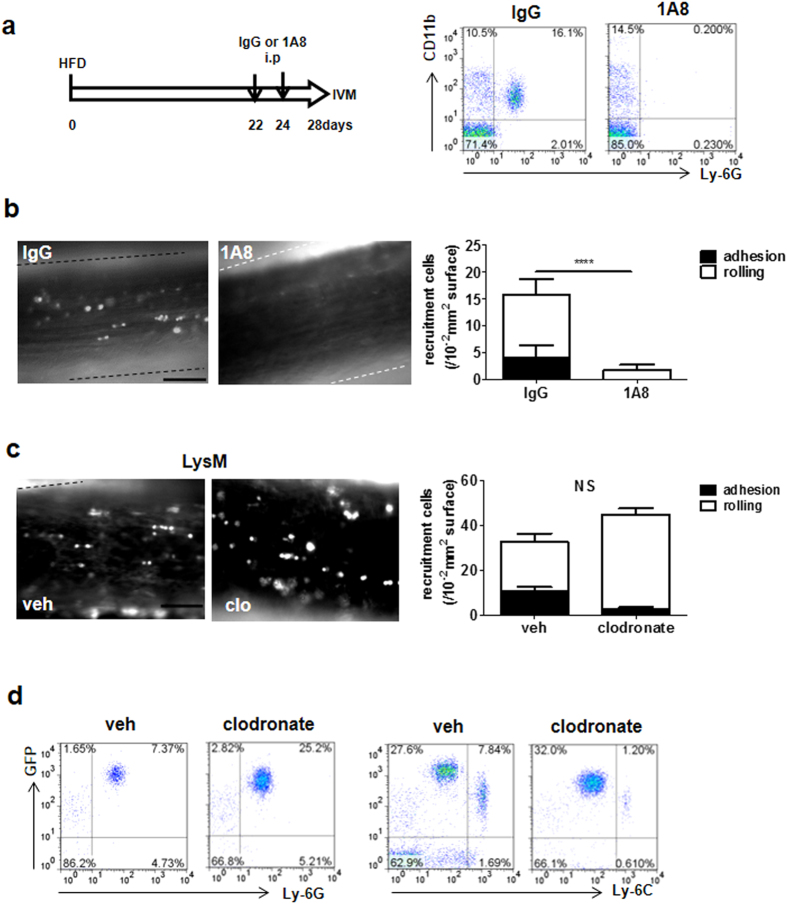
Neutrophils recruited in the femoral artery of wt mice by HFD. (**a**) Time schedule of the neutrophil depletion experiment. Wt mice were treated with the neutrophil depletion antibody 1A8 or the control IgG at 22 days and 24 days after starting HFD. Leukocyte recruitment was observed in these mice 28 days after HFD (left); 1A8 clearly diminished neutrophils in peripheral blood compared with IgG (right). (**b**) Leukocyte recruitment in the femoral artery of wt mice fed HFD treated with 1A8 (right image) or IgG (left image). Bar, 50 μm. The graph shows that leukocyte recruitment was significantly inhibited by neutrophil depletion (n = 4 and 4, respectively). Data are presented as the mean ± SEM. ****P < 0.0001 by unpaired; 2-tailed Student’s t test. **(c)** Monocyte depletion in the blood of LysM-eGFP mice using clodronate liposomes. The highest expression of GFP in peripheral leukocytes was observed in neutrophils, but monocytes were intermediately positive as well. Monocytes were then depleted using clodronate liposomes. Treatment of clodronate liposome-depleted GFP^+^Ly-6C^hi^ leukocytes as monocytes (right) in peripheral blood, but not GFP^+^Ly6G^+^ leukocytes as neutrophils (left). (**d**) Leukocyte recruitment in HFD-fed LysM-eGFP mice treated with clodronate liposomes to deplete monocytes. Clodronate liposomes were administered 24 h before observation. The image on the left shows the femoral artery treated with vehicle and that on the right shows the femoral artery treated with clodronate liposomes. Bar, 50 μm. Leukocyte recruitment did not change with monocyte depletion (graph) (n = 4 and 4, respectively). Data are presented as the mean ± SEM. NS (not significant) by unpaired; 2-tailed Student’s t test.

To confirm the involvement of neutrophils in HFD-induced leukocyte recruitment, IVM analysis was performed after neutrophils were depleted by injection of a Ly-6G–specific antibody, 1A8. Flow cytometry analysis of peripheral blood confirmed a depletion of neutrophils (Fig. 2a). HFD-induced leukocyte recruitment was significantly diminished when neutrophils were depleted (control IgG, 15.7 ± 0.89/10^4^-μm^2^ vessel surface, n = 4; 1A8, 0.50 ± 0.50/10^4^-μm^2^ vessel surface, n = 4, *P* < 0.0001; Fig. 2b and Supplementary Movie 9 and 10). When monocyte depletion was induced by intraperitoneal injection of clodronate liposomes in lysozyme M (LysM)-eGFP mice, in which neutrophils abundantly express GFP^13^ (Supplementary Fig. 2), leukocyte recruitment in the femoral artery did not change (Fig. 2c, and Supplementary Movie 11, 12), although the liposomes clearly caused monocyte depletion in peripheral blood (Fig. 2d). These results indicate that neutrophils, but not monocytes, were recruited to the femoral artery by 4 weeks of HFD.

### HFD increased C5a levels in the liver to mobilize and activate neutrophils

**Figure 3:**
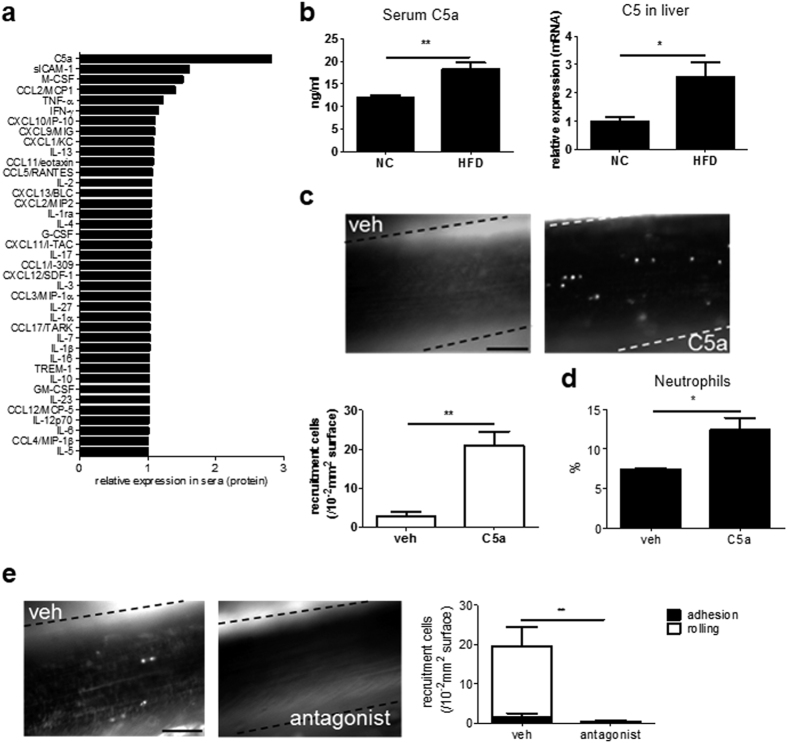
C5a levels increased by HFD and induced leukocyte recruitment. (**a**) Exhaustive cytokine array for sera from mice fed HFD. Cytokine array showed that C5a upregulates most in various cytokines by HFD. Sera pooled from eight mice were analyzed. **(b)** Feeding HFD for 4 weeks increased serum C5a levels (left) (n = 4 and 4, respectively). In addition, HFD increased C5 mRNA levels, the precursor of C5a, in the liver (right) (n = 8 and 8, respectively). Data are presented as the mean ± SEM. **P < 0.01 and *P < 0.05 by unpaired; 2-tailed Student’s t test. **(c)** Leukocyte recruitment in wt mice fed NC with recombinant mouse C5a after 1.5 h. Leukocyte recruitment was not detected when treated with vehicle (left panel), but observed when treated with C5a (right panel). Bar, 50 μm. C5a significantly increased leukocyte recruitment (n = 5 and 5, respectively). Data are presented as the mean ± SEM. **P < 0.01 by unpaired; 2-tailed Student’s t test. **(d)** 2 μg/kg of recombinant murine C5a was administered to wt mice fed NC. C5a induced neutrophilia for 1.5 hours (n = 4 and 4, respectively). Data are presented as the mean ± SEM. *P < 0.05 by unpaired; 2-tailed Student’s t test. **(e)** Leukocyte recruitment in wt mice fed HFD for 4 weeks with C5aR antagonist. Administration of C5aR antagonist diminished leukocyte recruitment induced by HFD (**P < 0.01) (n = 4 and 4, respectively). Data are presented as the mean ± SEM. **P < 0.01 by unpaired; 2-tailed Student’s t test. Bar, 50 μm.

To understand how HFD induces neutrophils to produce MCP-1, we measured the serum levels of various cytokines in mice fed HFD or NC for 4 weeks. As shown in Fig. 3a, the relative expression level of C5a in serum was the highest in HFD-fed mice (HFD 2.81, NC 1.00). This result was confirmed by ELISA for C5a as shown in Fig. 3b (HFD 18.15 ± 1.53 ng/ml, n = 4; NC 11.95 ± 0.52 ng/ml, n = 4; *P* < 0.01). C5a, an activated form of C5, is a potent neutrophil chemoattractant that is produced in the liver^14^,^15^,^16^ (Supplementary Fig. 3). Subsequent quantitative RT-PCR revealed that C5 levels in the liver were increased by HFD (HFD 2.57 ± 0.52, n = 8; NC 1.00 ± 0.13, n = 8; *P* < 0.05; Fig. 3b).

### Critical role of C5a in HFD-induced neutrophil recruitment

Because C5a can activate neutrophils, we conducted IVM analysis in mice treated with C5a in the absence of HFD. Administration of C5a alone markedly induced leukocyte recruitment in murine femoral arteries (vehicle 2.80 ± 1.16/10^4^-μm^2^ vessel surface, n = 5; C5a 21.00 ± 3.49/10^4^-μm^2^ vessel surface, n = 5; *P* < 0.01; Fig. 3c, Supplementary Movie 13, 14). Furthermore, C5a treatment significantly increased the number of peripheral neutrophils in wt mice (vehicle 7.38 ± 0.17%, n = 4; C5a 12.41 ± 1.55%, n = 4; *P* < 0.05; Fig. 3d). These data strongly suggest a critical role for C5a in HFD-induced neutrophil recruitment in the femoral artery. Furthermore, injection of C5a-receptor (C5aR) antagonist significantly diminished HFD-induced leukocyte recruitment (vehicle 19.50 ± 4.13/10^4^-μm^2^ vessel surface, n = 4; C5aR 0.25 ± 0.25/10^4^-μm^2^ vessel surface, n = 4; *P* < 0.01; Fig. 3e, Supplementary Movie 15, 16).

### HFD increased MCP-1 expression in neutrophils

**Figure 4:**
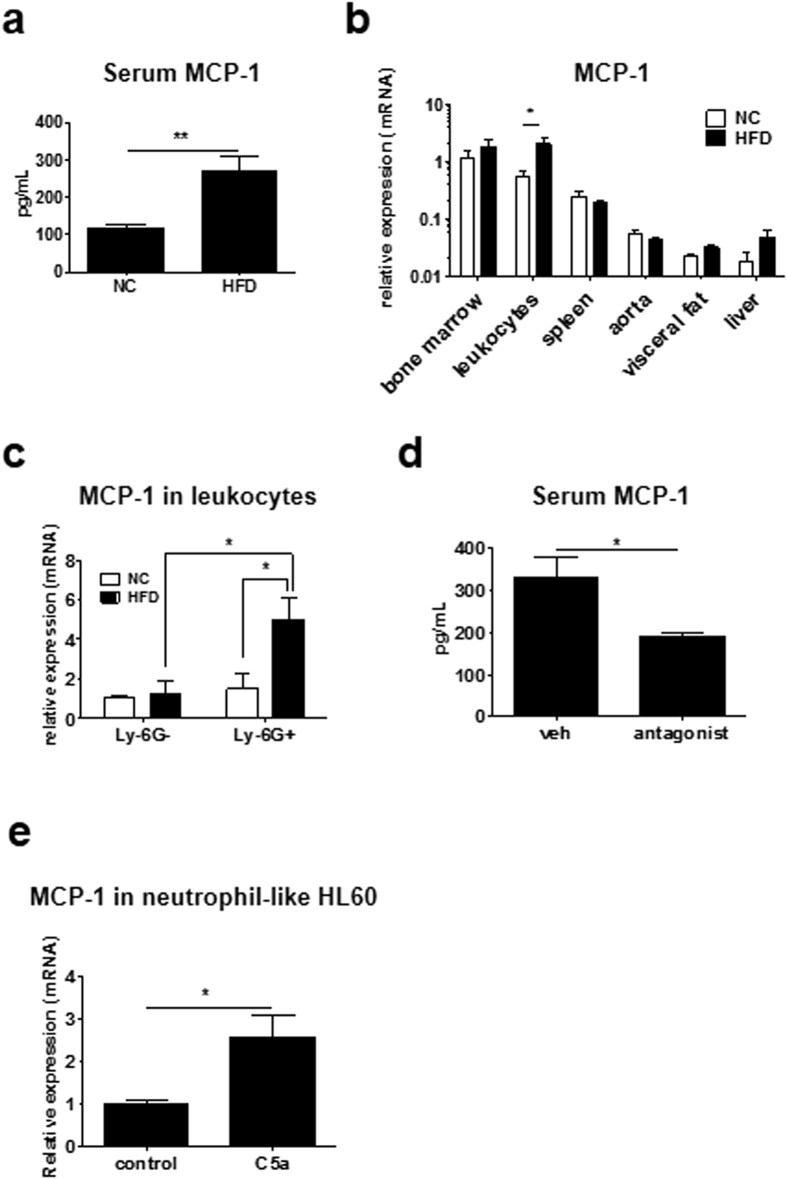
HFD upregulated MCP-1 expression in neutrophils. (**a)** HFD for 4 weeks significantly increased MCP-1 levels in sera of mice (n = 8 and 8, respectively). Data are presented as the mean ± SEM. **P < 0.01 by unpaired; 2-tailed Student’s t test. **(b)** MCP-1 mRNA levels in various tissues including bone marrow (n = 8 and 8, respectively), leukocytes (n = 4 and 5, respectively), spleen (n = 7 and 8, respectively), aorta (n = 6 and 8, respectively), visceral fat (n = 8 and 8, respectively) and liver (n = 8 and 7, respectively), of mice fed HFD or NC, involving 4 independent experiments. HFD significantly increased the MCP-1 level in circulating leukocytes. Data are presented as the mean ± SEM. *P < 0.05 by two-way ANOVA with Bonferroni’s post test. **(c)** HFD increased MCP-1 mRNA levels in neutrophils such as Ly-6G^+^ leukocytes, but not Ly-6G^−^ leukocytes (n = 3/each group), involving 4 independent experiments. Data are presented as the mean ± SEM. *P < 0.05 by two-way ANOVA with Bonferroni’s post test. **(d)** MCP-1 level in sera was measured by ELISA when C5aR antagonist was administrated in wt mice fed HFD for weeks. C5aR antagonist significantly decreased serum MCP-1 levels increased by HFD (n = 4 and 4, respectively). Data are presented as the mean ± SEM. *P < 0.05 by unpaired; 2-tailed Student’s t test. **(e)** mRNA level of MCP-1 in neutrophil-like HL-60 treated with 3nM recombinant C5a. C5a significantly increased MCP-1 expression level compared to control (n = 5 and 5, respectively), involving 4 independent experiments. Data are presented as the mean ± SD. *P < 0.05 by unpaired; 2-tailed Student’s t test.

Sustained HFD increases the serum MCP-1 levels^17^. Serum MCP-1 levels significantly increased as early as 4 weeks in HFD feeding compared with NC (HFD 268.1 ± 40.6 pg/ml, n = 8; NC 115.1 ± 10.43 pg/ml, n = 8; *P* < 0.01; Fig. 4a). When we measured the expression levels of MCP-1 in various tissues, the MCP-1 level was extremely high in leukocytes after 4 weeks of HFD (Fig. 4b). Because neutrophils did not express the putative MCP-1 receptor CCR2, we hypothesized that neutrophils were responsible for the production of MCP-1 in response to HFD. To confirm this hypothesis, the expression level of MCP-1 was separately measured in neutrophils (Ly-6G^+^) and monocytes/lymphocytes (Ly-6G^−^). As shown in Fig. 4c, HFD significantly upregulated MCP-1 expression in neutrophils but not in monocytes/lymphocytes (Ly-6G^−^, NC 1.00 ± 0.15, HFD 1.21 ± 0.71; Ly-6G^+^, NC 1.49 ± 0.73, HFD 5.01 ± 1.10, n = 3; *P* < 0.05). Furthermore, C5aR antagonist significantly decreased MCP-1 levels in sera of HFD-fed mice (vehicle 330.4 ± 49.92 pg/ml, n = 4; C5aR 189.8 ± 11.72 pg/ml, n = 4; *P* < 0.05; Fig. 4d). To demonstrate the direct influence of C5a on MCP-1 expression in neutrophils, we examined MCP-1 expression in HL60 differentiated to neutrophils under C5a treatment. MCP-1 expression under C5a treatment significantly increased in HL60 differentiated to neutrophils compared to control (control 1.00 ± 0.24, n = 5; C5a 2.58 ± 1.15, n = 5; *P* < 0.05; Fig. 4e). By contrast, MCP-1 expression level in undifferentiated HL-60 did not change between treatment of C5a and control (control 1.00 ± 0.24, n = 5; C5a 0.62 ± 0.27, n = 4; Supplementary Fig. 4), indicating that C5a influences differentiated neutrophils. These results demonstrate that C5a induce MCP-1 expression in neutrophils.

### Neutrophil-derived MCP-1 is required for HFD-induced increase of intimal leukocytes

**Figure 5:**
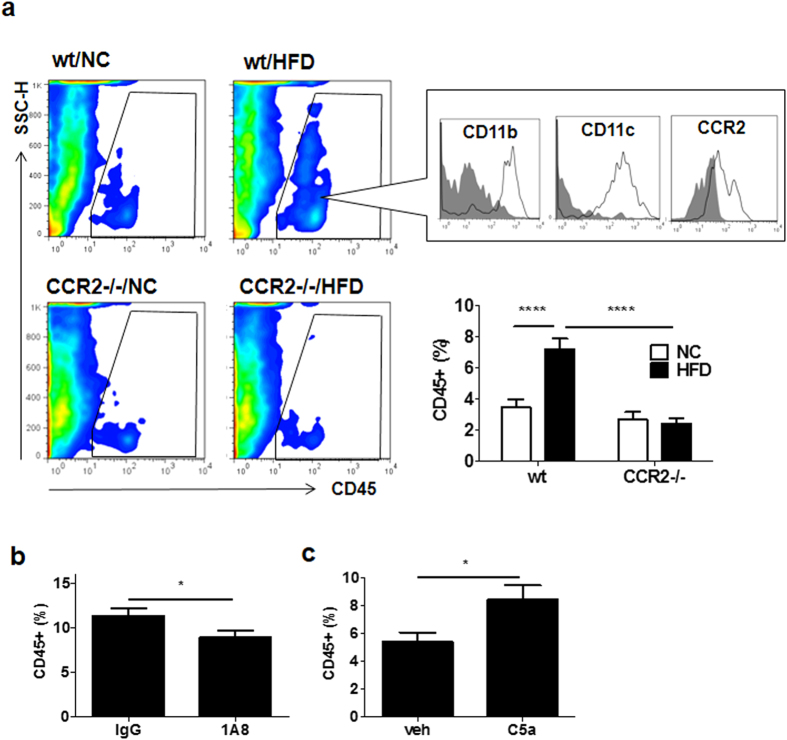
Neutrophils contributed to intimal CD45-positive leukocytes in vessels. (**a)** Flow cytometry for leukocytes in femoral artery of wt mice or CCR2^−/−^ mice fed NC or HFD. CD45^+^ cells were gated by the square (upper). Leukocytes in the intima significantly increased in wt mice fed HFD compared with NC. However, the CD45-positive cells did not change in CCR2^−/−^ mice between HFD and NC (n = 7/each group). These leukocytes were positive for CD11b, CD11c, and CCR2, involving 8 independent experiments. An isotype control antibody was used as the negative control (gray filled). Data are presented as the mean ± SEM. ****P < 0.0001 by two-way ANOVA with Bonferroni’s post test. **(b)** Flow cytometric analysis for single-cell suspension of femoral arteries of wt mice fed HFD for 4 weeks when neutrophils in peripheral blood were depleted by specific antibody. Neutrophil depletion significantly decreased the intimal leukocytes in the femoral artery (n = 6 and 6, respectively). Data are presented as the mean ± SEM. *P < 0.05 by unpaired; 2-tailed Student’s t test. **(c)** Flow cytometric analysis for single-cell suspension of the femoral arteries of wt mice treated with C5a for 4 weeks. C5a significantly increased leukocytes in the femoral artery (n = 8 and 7, respectively). Data are presented as the mean ± SEM. *P < 0.05 by unpaired; 2-tailed Student’s t test.

Although we are able to determine a critical role for neutrophils in HFD-induced vascular inflammation, it is unclear whether recruited neutrophils actually play a role in subsequent vascular inflammation in the intimal area. To monitor the vascular inflammation in intimal and medial areas, a flow cytometry analysis for single-cell suspension of femoral artery specimen was performed. On feeding HFD for 4 weeks, number of CD45-positive cells significantly increased in the femoral artery than on feeding NC (HFD 7.21 ± 0.67%, n = 7; NC 3.47 ± 0.50%, n = 7; *P* < 0.0001; Fig. 5a). Disruption of the MCP-1/CCR2 signal using CCR2-deficient (CCR2^−/−^) mice exhibited a similar reduction of HFD-associated CD45-positive cells in femoral arteries (HFD 2.40 ± 0.38%, n = 7; NC 2.62 ± 0.51%, n = 7; Fig. 5a). As shown in Fig. 5a, majority of the CD45-positive cells were positive for CD11b, CD11c. To confirm the importance of neutrophils in these intimal leukocytes, neutrophil depletion was performed for wt mice fed HFD for 4weeks. As shown in Fig. 5b, neutrophil depletion significantly diminished HFD-induced intimal CD45-positive cells in femoral arteries (control IgG 11.38 ± 0.82%, n = 6; 1A8 8.96 ± 0.69%, n = 6; *P* < 0.05). Furthermore, when wt mice were subjected to murine recombinant C5a for 28 days, the number of intimal CD45-positive cells in the femoral artery was significantly increased compared with vehicle (vehicle 5038 ± 0.69%, n = 7; C5a 8.41 ± 1.04%, n = 8; *P* < 0.05; Fig. 5c). These results indicated that the up-regulation of serum MCP-1, peripheral neutrophils and serum C5a play an important role in the increase of intimal CD45-positive cells by HFD.

## Discussion

Leukocyte recruitment is a key mechanism of vascular inflammation. Therefore, critical observation of leukocyte recruitment in large vessels in the initial phase of vascular inflammation is particularly important. However, the observation of large-diameter vessels using intravital microscopy is technically difficult and non-physiological vessel positions may affect the data. In this study, we could capture leukocyte recruitment in the large artery of mice fed HFD from as early as 4 weeks. To the best of our knowledge, this is the first observation that such leukocyte recruitment is induced by HFD alone. Previous studies indicated that prolonged ingestion of HFD initiates metabolic disorders such as obesity and type 2 diabetes^18^,^19^. According to a recent observation by Progatzky *et al*., dietary cholesterol induces acute inflammation in intestine in zebra fish^20^. Our study further expands the acute effect of HFD toward vascular inflammation in a large vessel. Although we do not know the impact of a high-cholesterol diet or HFD on vascular inflammation, future study will focus on the effect of different lipid components on observed vascular inflammation. In this study, neutrophils were found to play a pivotal role in the initial phase of HFD-induced vascular inflammation.

Previous studies have emphasized on the role of monocytes, macrophages, and lymphocytes or their subsets in HFD-induced chronic inflammation in adipose tissues^21^,^22^,^23^,^24^ and atherosclerosis^25^,^26^,^27^. However, our results suggest that neutrophils play a dominant role in the phase of acute inflammation induced by HFD. Neutrophils are considered as the first immune cells to respond to inflammatory stimuli and maintain and amplify the inflammatory state by helping to recruit macrophages and lymphocytes. The importance of neutrophils in the pathogenesis of metabolic disorders recently attracted considerable attention. A previous study demonstrated that neutrophil-derived elastase causes cellular insulin resistance in HFD-fed mice^28^. Although the precise mechanisms of how HFD activates neutrophils are unknown, the elevation of C5a levels plays a primary role in this process.

C5a is a strong chemoattractant and activator of neutrophils^15^,^16^. Previous studies have extensively studied its role in acute inflammation such as that in case of sepsis, rheumatoid arthritis, and inflammatory bowel disease^15^,^29^,^30^,^31^. However, its effect on metabolic disorders is not understood and we are the first to demonstrate a link between HFD and C5a activation.

The complement system plays a central role in the innate immune response and eliminates pathogens, tumor cells, and apoptotic self-cells^32^,^33^. C5a is a member of the alternative pathway under the influence of C1q, C2b, C4b, C3b, and the decay-accelerating factor (CD55)^15^. Notably, expression levels of these factors in the liver of mice fed with HFD for 4 weeks did not significantly change compared with those fed with NC (Supplementary Fig. 5). These results suggest that HFD primarily contributes to the upregulation of C5.

HFD increases serum MCP-1 levels in wt mice. Although previous reports have suggested an important role for adipose tissue as a source of MCP-1 by HFD, we failed to demonstrate an increase of MCP-1 mRNA levels from visceral fat tissue with 4 weeks of HFD (Fig. 4b). Nevertheless, we observed an HFD-associated increase of MCP-1 mRNA levels in leukocytes. Considering the major roles of MCP-1 in vascular inflammation including that in case of atherosclerosis, the dominant contribution of neutrophils as a source of MCP-1 will provide them great importance. Administration of C5a alone can induce leukocyte recruitment in the femoral artery (Fig. 3c) and increase peripheral neutrophil counts (Fig. 3d). C5a also induces MCP-1 production in neutrophils (Fig. 4e), suggesting causative roles of C5a and MCP-1 in acute phase inflammation induced by HFD.

MCP-1 appears to play a role in the increase of intimal CD45-positive cells by HFD which is abolished in CCR2^−/−^ mice (Fig. 5a). These results show that MCP-1/CCR2 signal mediate the increase of the CD45-positive cells in intima by HFD. These intimal cells are positive for CD11b, CD11c, and CCR2, suggesting that they are of monocyte lineage. The number of CD45-positive cells were reduced by neutrophil depletion (Fig. 5b) and increased by C5a treatment (Fig. 5c), suggesting that these intimal CD45-positive cells are under the control of neutrophils via C5a. However, the characteristics of these CD45-positive cells remain to be studied.

C5a is involved in the development of abdominal aortic aneurysm^34^ and genetically engineered atherosclerosis^35^. In this study, we found that C5aR antagonist markedly inhibited leukocyte recruitment in the femoral artery (Fig. 3e) and reduced serum MCP-1 level (Fig. 4d) in HFD-fed mice. Our results may expand a novel therapeutic approach toward HFD-induced metabolic disorders.

**Figure 6:**
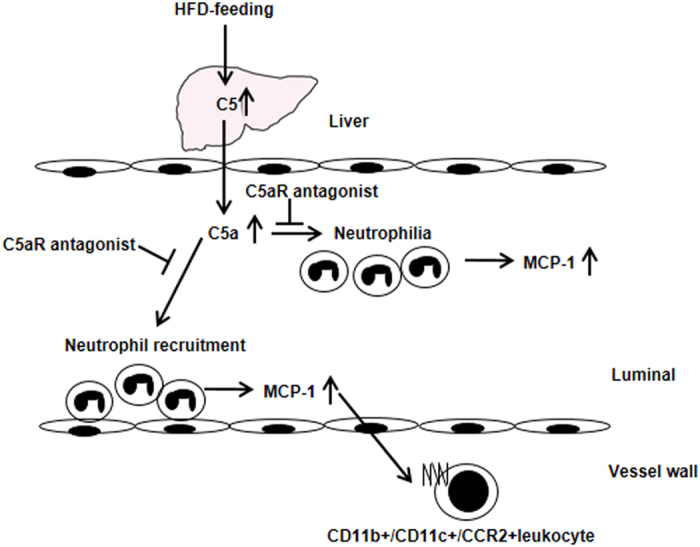
Proposed mechanisms of vascular inflammation by HFD. HFD increased C5 expression levels in the liver and serum C5a levels. C5a induced neutrophilia and neutrophil recruitment in the femoral artery. Neutrophil recruitment contributed to the CD11c^+^ intimal leukocytes in the femoral artery.

In conclusion, we confirmed that HFD alone can initiate neutrophil-mediated acute leukocyte recruitment via hepatic induction of C5a. This luminal step proceeds to intima of leukocytes via the MCP-1/CCR2 axis. Our finding may shed a light on previously unrecognized mechanism of vascular inflammation in acute phase of chronic inflammatory conditions such as atherosclerosis induced by HFD (Fig. 6).

## Materials and Methods

### Animals

Seven-week-old male wt C57BL/6 mice were obtained from Charles River Laboratories Japan, Inc., and 7-weeks-old male LysM-eGFP mice were a kind gift from Dr. Y. Inomata (Department of Transplantation and Pediatric Surgery, Postgraduate School of Medical Sciences, Kumamoto University, Kumamoto, Japan). Seven-week-old male CCR2^−/−^ mice used in this study were a kind gift from Dr. K. Egashira (Department of Cardiovascular Medicine, Graduate School of Medical Sciences, Kyushu University, Fukuoka, Japan). The mice were fed the respective diet and water *ad libitum*. The experiments adhered to the APS Guiding Principles in the Care and Use of Animals and were approved by the Ethical Committee for Animal Experimentation of Tokyo Medical and Dental University and methods were carried out in accordance with their approved guidelines.

### IVM

Wt, LysM-eGFP, and CCR2^−/−^ mice were started on HFD (1.25% cholesterol and 20% fat; CLEA Japan, Inc. and Sankyo Labo Service Corporation, Inc, respectively) or normal chow (NC; CE-2, CLEA Japan, Inc.) at the age of 7 weeks. IVM was performed on the femoral arteries of wt mice fed HFD or NC for 1, 2, 4, or 8 weeks and LysM-eGFP and CCR2^−/−^ mice fed HFD for 4 weeks. Observation of leukocyte recruitment in the femoral artery using IVM has been described previously^36^,^37^. In brief, mice were anesthetized with pentobarbital and mechanically ventilated so as to maintain a normal blood pH. For observation under normal physiological conditions, rectal temperature was maintained at 36.0 °C–37.0 °C with a heating pad and an infrared heat lamp during the experiment. The legs were shaved, and the femoral artery and vein were exposed. After injection of Rhodamine 6G chloride ([Invitrogen, Carlsbad, CA, USA; 0.3 mg mg/kg-1 in 300 μl of phosphate buffered saline without calcium and magnesium: PBS (−)] into the right femoral vein, the left femoral artery at the level of the epigastric branch was visualized with a fluorescent microscope (BX51WI, Olympus) equipped with a water immersion objective (20×). Through observation, the warmed PBS (−) was supplied to the tissue. All movies were recorded for 1 min with 5 frames per second using a computer-assisted image analysis program (Meta Morph). The parameters used to characterize the adhesive or rolling interactions of leukocytes have previously been described in detail^36^,^37^.

### Flow cytometry and leukocyte count in peripheral blood

Peripheral bloods were harvested by cardiac puncture. Rat anti-mouse CD11b-FITC, rat anti-mouse CD45-PerCP, rat anti-mouse Ly6G-APC, rat anti-mouse Ly6C-APC, rat anti-mouse CD3-PE, or rat anti-mouse CD19-PE (Biolegend, Inc.) in 1.8 ml of Lysing Buffer (BD Biosciences) were added to 200 μl of blood and incubated for 15 min at room temperature in the dark. After washing, cells were fixed with 1% paraformaldehyde in PBS (−) and analyzed by a method similar to that for the femoral artery. Flow cytometry analyses were performed for 10,000 cells of single-cell suspension using BD FACS Calibur, and data were analyzed with FlowJo7.6. Neutrophils were gated at CD45^+^Ly-6G^+^CD11b^+^. Monocytes were gated at CD45^+^Ly-6C^hi^CD11b^+^ and lymphocytes were gated at CD45^+^CD3^+^ and CD45^+^CD19^+^. The leukocyte count was determined using XT-2000iV (Sysmex, Kobe, Japan).

### Neutrophil depletion

As shown Fig. 2A, wt mice were intraperitoneally injected with 250 μg of rat anti-Ly6G antibody (clone 1A8, Bio X cell) at 22 days and 24 days after feeding HFD^38^,^39^. As a control group, normal rat IgG2a isotype control (R&D Systems, Inc.) was treated. At 28 days, IVM was performed on these mice. The blood obtained was analyzed using flow cytometry and MCP-1 levels in the isolated sera were measured using ELISA.

### Monocyte depletion

Neutrophils of LysM-eGFP mice are dominantly high in GFP fluorescence level. However, even GFP of approximately 1/10 in neutrophils express in monocytes^13^ (Supplementary Fig. 2). We then demonstrated leukocyte recruitment in LysM-eGFP mice fed HFD for 4 weeks under monocyte depletion using clodronate liposomes. LysM-eGFP mice fed HFD for 28 days were administered 200 μl of clodronate liposomes (FormuMax Scientific, Inc.) by intravenous injection and IVM was performed after 24 h.

### Cytokine array for sera from HFD- or NC-fed mice

Peripheral blood was harvested by cardiac puncture from wt mice fed HFD or NC for 4 weeks and serum was isolated. Sera was pooled from eight mice and applied to Mouse Cytokine Array (R&D Systems, Inc.) according to the manufacturer’s protocol. The chemiluminescent images were captured, and the pixel intensity in each spot of the array was analyzed by a luminescent image analyzer LAS-1000 (Fuji Photo Film Co., Ltd.). The relative intensity of each cytokine from HFD mice was expressed proportional to the intensity of that cytokine in NC-fed mice as 1.00.

### Analysis of chemokines and other cytokines in sera

Blood was obtained by cardiac puncture from wt mice fed HFD for 4 weeks and the serum was isolated. MCP-1 and C5a levels in the sera were quantified by ELISA. In brief, 96-well microplates coated with captured antibody, anti-mouse MCP-1 antibody (R&D Systems, Inc.), or anti-mouse C5a antibody (R&D Systems, Inc.) were blocked by 5% bovine serum albumin in PBS (−) for 1 h at room temperature followed by reaction of the samples for 2 h. The microplates were washed three times with 0.02% Tween-20 in PBS (−) and incubated with 100 μl of antibody for detection for 2 h. After washing, 100 μl of HRP-streptavidin (R&D Systems, Inc.) was added to the microplates and incubated for 30 min, followed by washing. The immunoreaction was developed by adding substrate (KPL, Inc.) and 0.6N H_2_SO_4_ solution was added to stop the reaction. The absorbance at 450 nm was measured and the concentration in the samples was calculated using the standard curves.

### Quantitative RT-PCR for MCP-1 and C5 in tissues and neutrophils

Wt mice fed NC or HFD for 4 weeks were perfused from the left ventricle with PBS (−) to harvest aorta, liver, spleen, bone marrow, and epididymal fat. Circulating leukocytes were isolated from the hemolysate. Total RNA was isolated from each tissue using RNeasy (QIAGEN). Peripheral bloods were harvested by cardiac puncture and blood from for mice was pooled. Neutrophils were isolated from the pooled blood of three wt mice fed NC or HFD for 4 weeks using an anti-Ly-6G microbeads kit (Milterny Biotec GmbH) according to the manufacture’s protocol. Total RNA was isolated from the obtained neutrophils using ReliaPrepTM RNA Cell Miniprep System (Promega Corporation). First-strand cDNA was synthesized using transcriptase (Takara Bio Inc.) and quantitative PCR was performed using the Thermal Cycler Dise® Real Time System (Takara Bio Inc.) with KAPA STBR FAST Universal qPCR Kit (Kapa Biosystems). The relative expression level was calculated by standard curve methods and ΔΔCt methods using 18S ribosomal RNA as the internal control. The oligonucleotide primers for the experiments were: murine MCP-1: 5′-CTGTGCTCAGAGCTTTCAAC-3′ and 5′-TCTCCCTTTGCAGAACTCAG-3′; human MCP-1: 5′-CCAAGCAGAAGTGGGTTCAG-3′ and 5′-CTTGGGTTGTGGAGTGAGTG-3′; C5: 5′-TGAGCGTCATGTCCTACAGA-3′ and 5′-CACCTGTCCAAGCACTCTCA-3′; and 18S ribosomal RNA: 5′-GTAACCCGTTGAACCCCATT-3′ and 5′-CCATCCAATCGGTAGTAGCG-3′.

### Administration of C5a to mice

Wt mice were administered recombinant mouse C5a (2 μg/kg, ProSpec-Tany TechnoGene Ltd.) by intravenous injection. After 90 min, leukocyte recruitment was observed using IVM and blood was harvested for flow cytometry analysis.

### Administration of C5aR antagonist to HFD-fed mice

HFD-fed wt mice were administered C5aR antagonist (0.3 mg/kg/day, KareBay Biochem, Inc.) or vehicle [PBS (−)] by intraperitoneal injection. After 4 weeks, leukocyte recruitment in these mice was observed and MCP-1 levels in the sera were measured by ELISA.

### Treatment of C5a to HL-60 differentiated neutrophils

To differentiate to neutrophil-like, HL-60 cells (ATCC) were cultured in RPMI1640 containing 10% fetal bovine serum and 1.3% DMSO for 5days (Millius and Weiner, 2009). Differentiated cells were treated with 3nM of recombinant human C5a (PeproTech, inc). After 1hour, these cells were harvested and isolated mRNA to analyze MCP-1 expression by quantitative RT-PCR.

### Flow cytometry analysis for leukocytes in femoral arteries

Flow cytometry for single-cell suspension of femoral arteries was performed to analyze adherent or infiltrated leukocytes in vessel. Wt mice fed NC or HFD for 4 weeks and CCR2^−/−^ mice fed HFD for 4 weeks were perfused from the left ventricle with PBS (−). Femoral arteries were obtained carefully and vessels from two mice were pooled. The collected vessels were digested by 125 U/ml collagenase type XI, 60 U/ml hyaluronidase type I-s, 60 U/ml DNase1, and 450 U/ml collagenase type I (all enzymes were obtained from Sigma-Aldrich) in PBS (−) containing 20 mM HEPES at 37 °C for 1 h with shaking^40^. The single cell suspensions were filtered with a 36 μm-nylon mesh and centrifuged at 2 *g* for 5 min at 4 °C. The pellets were reacted with rat anti-mouse CD11b-FITC (Biolegend, Inc.), rat anti-mouse CD11c-PE(Biolegend, Inc.), rat anti-mouse CD45-PerCP (Biolegend, Inc.), and anti-mouse/rat CCR2-APC (R&D Systems Inc.) at 4 °C for 20 min. For washing, the cell suspensions were added to 2 mM EDTA in PBS (−) and centrifuged at 2 *g* for 5 min at 4 °C. After fixation of the cells with 1% paraformaldehyde in PBS (−), flow cytometry analysis was performed.

### Statistical analysis

Data are expressed as the mean value ± SEM. Two-way ANOVA with Bonferroni’s post test or unpaired two-tailed Student’s t test was used to estimate statistical significance, with a value of *P* < 0.05 considered statistically significant.

## Additional Information

**How to cite this article**: Osaka, M. *et al*. Critical role of the C5a-activated neutrophils in high-fat diet-induced vascular inflammation. *Sci. Rep.* **6**, 21391; doi: 10.1038/srep21391 (2016).

## Supplementary Material

Supplementary Information

supplementary movie 1

supplementary movie 2

supplementary movie 3

supplementary movie 4

supplementary movie 5

supplementary movie 6

supplementary movie 7

supplementary movie 8

supplementary movie 9

supplementary movie 10

supplementary movie 11

supplementary movie 12

supplementary movie 13

supplementary movie 14

supplementary movie 15

supplementary movie 16
