# Supplementary material for: Critical role of the C5a-activated neutrophils in high-fat diet-induced vascular inflammation
Source: Sci Rep. 2016 Feb 19;6:21391. doi: 10.1038/srep21391 (PMC4759545; doi:10.1038/srep21391)
Supplement: Supplementary Information [file srep21391-s1.pdf]

Critical role of the C5a-activated neutrophils in high-fat diet-induced vascular inflammation

Mizuko Osaka[1], Shunsuke Ito[1], Masaki Honda[2], Yukihiro Inomata[2], Kensuke Egashira[3], and Masayuki Yoshida[1]

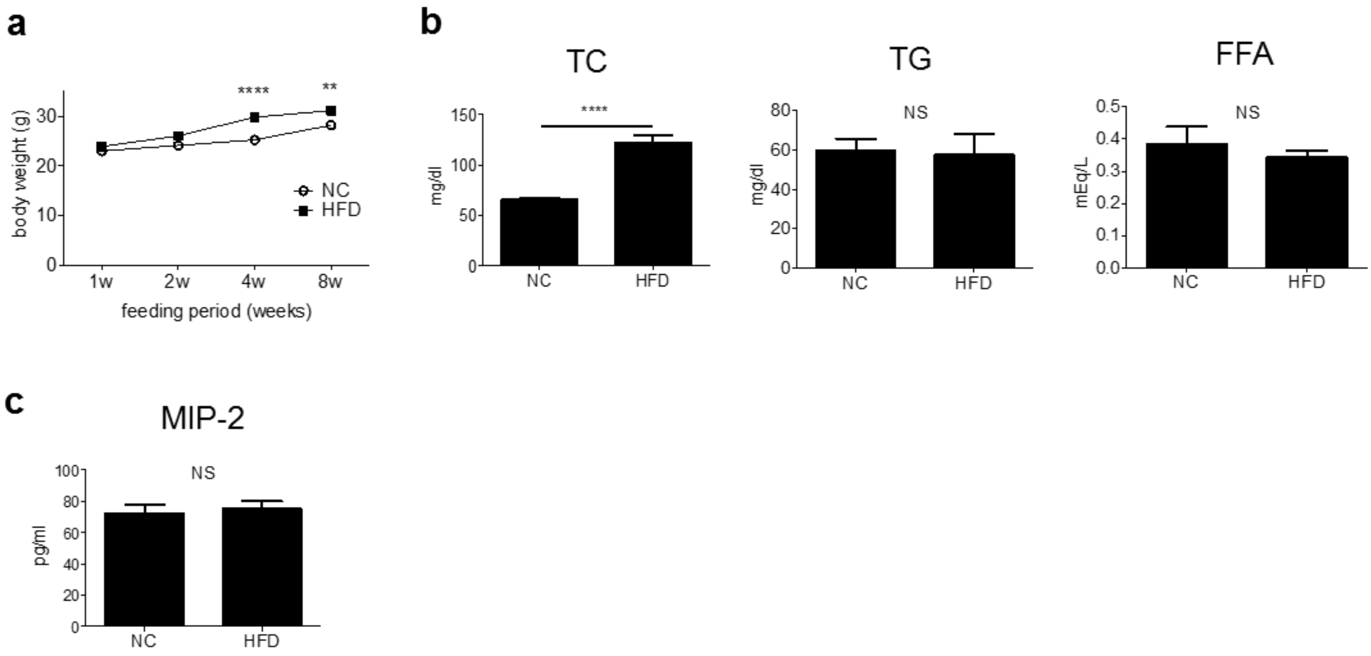

Supplementary Figure1. Physiological parameters of wt mice fed HFD or NC. (a) Body weight of wt mice fed HFD or NC for 1 (n=12 and 9, respectively), 2 (n=12 and 12, respectively), 4 (n=12 and 15, respectively), 8weeks (n=12 and 15, respectively) (n=8 and 7, respectively). Data are presented as the mean ± SEM. ****P < 0.0001 and **P < 0.01 by two-way ANOVA with Bonferroni's post test. **(b)** Serum lipid profile which is total cholesterol (n=8 and 8, respectively), triglyceride (n=7 and 6, respectively) and free fatty acid (n=4 and 4, respectively) of mice fed HFD or NC for 4weeks. Data are presented as the mean ± SEM. ****P < 0.0001 and NS (not significant) by unpaired; 2-tailed Student's t test. **(c)** MIP-2 level in sera of mice fed HFD or NC for 4weeks (n=12 and 12, respectively). Data are presented as the mean ± SEM. NS (not significant) by unpaired; 2-tailed Student's t test.

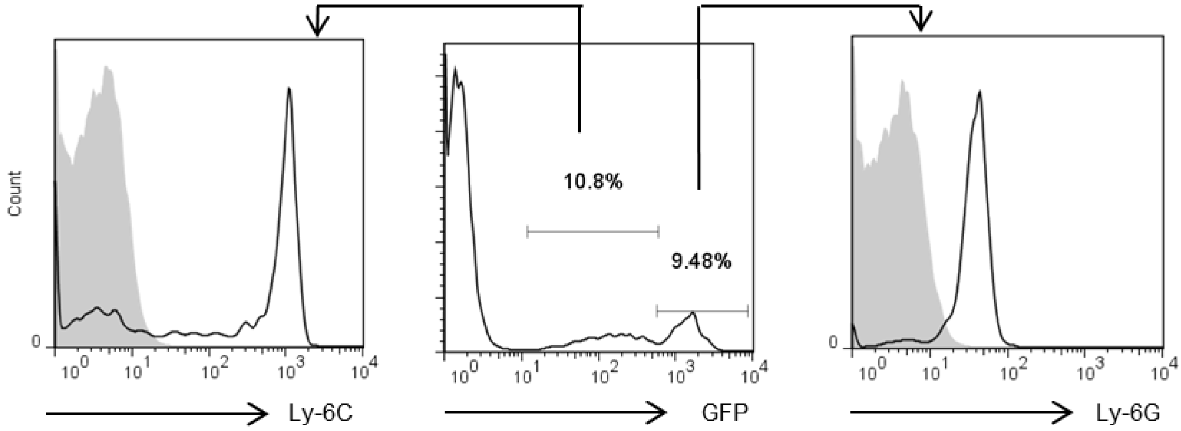

Supplementary Figure 2. GFP expression level in peripheral leukocytes. The highest expression of GFP in peripheral leukocytes is neutrophil, but monocytes were middle-positive. Peripheral leukocytes were stained with anti-Ly-6C antibody or anti-Ly-6G antibody (white). Leukocytes stained with control IgG were used as negative control (gray filled).

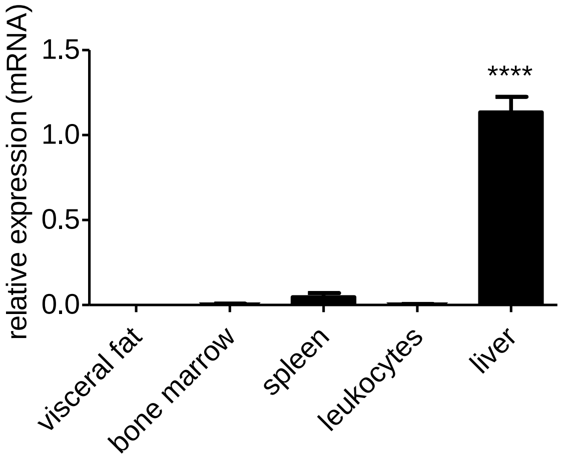

Supplementary Figure 3. Relative mRNA expression level of C5 which is precursor of C5a, in each tissue. Relative mRNA expression level of C5 in visceral fat, bone marrow, spleen, circulating leukocytes and liver of wt mice. Expression level of C5 in liver significantly is prominently high compared to other tissue (n=4/each group). Data are presented as the mean ± SEM. ****P < 0.0001 by two-way ANOVA with Bonferroni's post test.

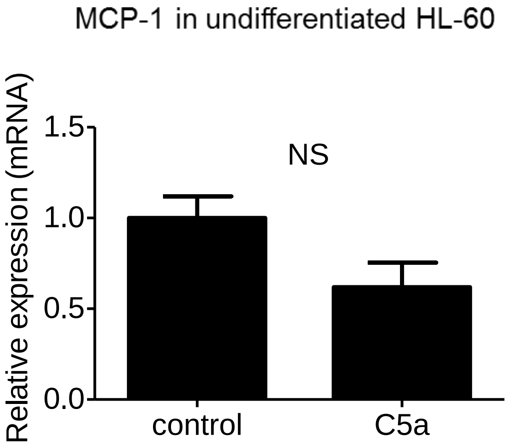

Supplementary Figure 4. Relative mRNA level of MCP-1 in undifferentiated HL-60 treated with recombinant C5a. Treatment of C5a (3nM) to undifferentiated HL-60 did not change relative mRNA level of MCP-1 in these cells compared to control, involving 4 independent experiments (n=4 and 4, respectively). Data are presented as the mean ± SEM. NS (not significant) by unpaired; 2-tailed Student's t test.

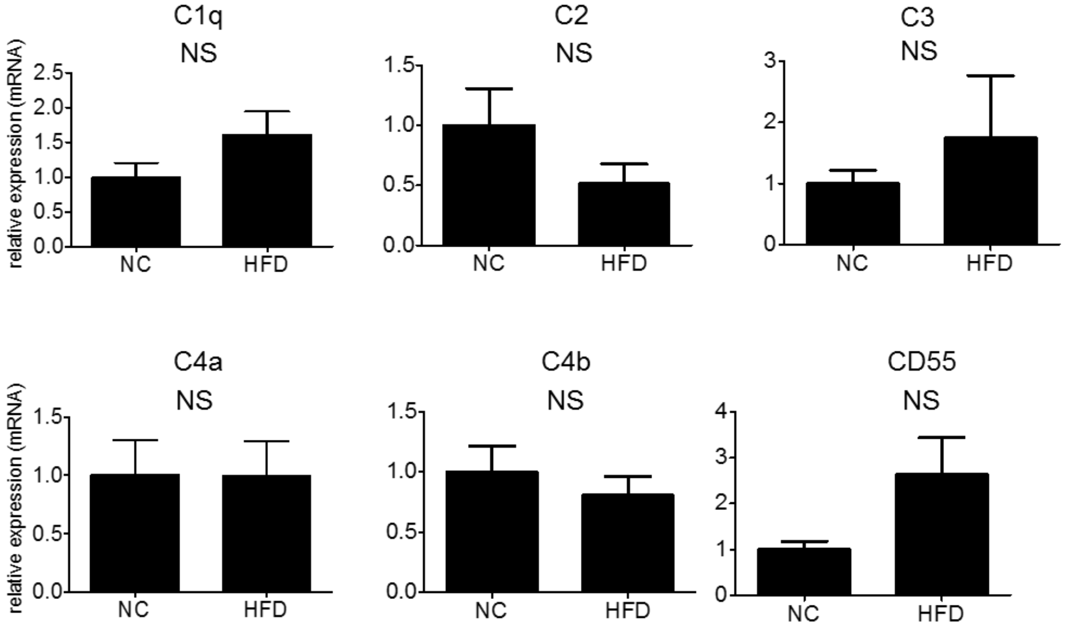

Supplementary Figure 5. Relative mRNA expression level of each complement components and decay accelerating factor (CD55) in liver of wt mice fed HFD or NC. The relative mRNA level of C1q, C2, C3, C4a, C4b and CD55 didn't change between HFD and NC, involving 4 independent experiments (n=8/each group). Data are presented as the mean ± SEM. NS (not significant) by unpaired; 2-tailed Student's t test.

Supplementary Movie Legend

Supplementary Movie 1. Movie of leukocyte recruitment in femoral artery of wt mouse fed HFD for 1weeks. Images were captured by intravital microscopy system. Leukocytes of those mice did not interact with vessel surface.

Supplementary Movie 2. Movie of leukocyte recruitment in femoral artery of wt mouse fed NC for 1weeks. This movie is control for Supplementary Movie 1.

Supplementary Movie 3. Movie of leukocyte recruitment in femoral artery of wt mouse fed HFD for 2weeks.

Supplementary Movie 4. Movie of leukocyte recruitment in femoral artery of wt mouse fed NC for 2weeks. This movie is control for Supplementary Movie 3.

Supplementary Movie 5. Movie of leukocyte recruitment in femoral artery of wt mouse fed HFD for 4weeks. Significant leukocyte recruitment was observed when compared with NC.

Supplementary Movie 6. Movie of leukocyte recruitment in femoral artery of wt mouse fed NC for 4weeks. This movie is control for Supplementary Movie 5.

Supplementary Movie 7. Movie of leukocyte recruitment in femoral artery of wt mouse fed HFD for 8weeks.

Supplementary Movie 8. Movie of leukocyte recruitment in femoral artery of wt mouse fed NC for 8weeks. This movie is control for Supplementary Movie 7.

Supplementary Movie 9. Movie of leukocyte recruitment in femoral artery of HFD-fed wt mouse when neutrophils were depleted by anti-Ly-6G antibody, 1A8. Neutrophil depletion clearly diminished leukocyte recruitment by HFD-feeding.

Supplementary Movie 10. Movie of leukocyte recruitment in femoral artery of HFD-fed wt mouse treated with control IgG for 1A8. Neutrophil depletion clearly diminished leukocyte recruitment by HFD-feeding. This movie is control for Supplementary Movie 9.

Supplementary Movie 11. Movie of leukocyte recruitment in femoral artery of HFD-fed LysM-eGFP mouse when peripheral monocytes were depleted by clodronate liposome. Monocyte depletion did not change leukocyte recruitment by HFD-feeding.

Supplementary Movie 12. Movie of leukocyte recruitment in femoral artery of HFD-fed LysM-eGFP mouse treated with PBS(-). This movie is control for Supplementary Movie11.

Supplementary Movie 13. Movie of leukocyte recruitment in femoral artery of NC-fed wt mouse treated with murine recombinant C5a. Administration of C5a significantly induced leukocyte recruitment.

Supplementary Movie 14. Movie of leukocyte recruitment in femoral artery of NC-fed wt mouse treated with PBS(-) as vehicle of C5a. Administration of C5a significantly induced leukocyte recruitment. This movie is control for Supplementary Movie13.

Supplementary Movie 15. Movie of leukocyte recruitment in femoral artery of HFD-fed wt mouse treated with C5aR antagonist. Administration of C5aR antagonist significantly inhibited leukocyte recruitment.

Supplementary Movie 16. Movie of leukocyte recruitment in femoral artery of HFD-fed wt mouse treated with PBS(-) as vehicle of C5aR antagonist. This movie is control for Supplementary Movie15.
